# Interactions of CFTR and Arylsulfatase B (ARSB; N-acetylgalactosamine-4-sulfatase) in Prostate Carcinoma

**DOI:** 10.3390/ijms26094350

**Published:** 2025-05-03

**Authors:** Sumit Bhattacharyya, Joanne K. Tobacman

**Affiliations:** 1 Jesse Brown VA Medical Center, Chicago, IL 60612, USA; ramabha@uic.edu; 2 Department of Medicine, College of Medicine, University of Illinois Chicago, Chicago, IL 60612, USA

**Keywords:** cystic fibrosis, CFTR, Arylsulfatase B, chondroitin sulfate, glycosaminoglycans, prostate cancer

## Abstract

Defective CFTR (cystic fibrosis transmembrane conductance regulator) is pathognomonic for cystic fibrosis (CF), which is characterized by an accumulation of tenacious secretions in pulmonary airways, as well as by abnormal ductal secretions in other organs, including the pancreas and prostate. The advent of CFTR modulating therapies has markedly improved the clinical status and survival of CF patients, primarily attributable to improved lung function. Previous publications reported that a decline in CFTR function was associated with a decline in activity and expression of the enzyme N-acetylgalactosamine-4-sulfatase (Arylsulfatase B; ARSB). ARSB removes 4-sulfate groups from N-acetylgalactosamine 4-sulfate residues and is required for the degradation of chondroitin 4-sulfate (chondroitin sulfate A) and dermatan sulfate, two sulfated glycosaminoglycans which accumulate in cystic fibrosis. Declines in both ARSB and in CFTR have been associated with the development of malignancies, including prostate malignancy. The experiments in this report show that similar effects on invasiveness are present when either CFTR or ARSB is inhibited in human prostate epithelial cells, and these effects resemble findings detected in malignant prostate tissue. The effects of CFTR inhibition are reversed by treatment with recombinant human ARSB in prostate cells. These results suggest that treatment by rhARSB may benefit patients with cystic fibrosis and prostate cancer.

## 1. Introduction

In recent years, life expectancy for patients with cystic fibrosis has markedly improved, due to innovative, effective treatment by CFTR (cystic fibrosis transmembrane conductance regulator) modulators. A consequence of improved life expectancy appears to be an increased incidence of several malignancies, which are occurring at increased frequency relative to the non-CF population. Increased occurrence of pancreatic, intestinal, lung, and prostatic malignancies has been reported in association with CF [1,2,3,4,5]. The mechanisms by which genetic mutations of CFTR might lead to increased malignant transformation have not been elucidated.

In previous publications, we have reported that a decline in activity and expression of the enzyme N-acetylgalactosamine 4-sulfatase (Arylsulfatase B; ARSB) occurs in association with defective CFTR in circulating leukocytes of patients with CF [6] and in CF cell lines [7]. Consistent with these observed declines in ARSB when CFTR was defective, treatment by CFTR modulators or CFTR gene therapy increased ARSB activity and expression in bronchial epithelial cells [7]. The CFTR-induced increase in CREB (cAMP-responsive element binding protein) and the CREB-mediated effect on the ARSB promoter were implicated as the underlying mechanism by which CFTR regulated ARSB gene expression in normal human primary bronchial epithelial cells [7]. The effects of defective CFTR on chloride were also considered in relation to inhibition of ARSB activity, since high chloride concentration inhibited ARSB activity in human kidney cells in the renal tissue of salt-sensitive rats on a high sodium chloride diet [7]. Additionally, the formylglylcine-modifying enzyme, which is required for the post-translational modification and activation of ARSB, can be inhibited by chloride ions [8], suggesting another mechanism by which CFTR regulation of chloride might impair ARSB function. In the remote past, declines in CFTR and ARSB may have provided a selective advantage to humans against malaria, since malarial-infected red blood cells bind less well to the endothelium when chondroitin 4-sulfate is more sulfated [9,10,11], implying a survival benefit against malaria from ARSB deficiency.

In contrast to the potential benefit of lower ARSB and enhanced chondroitin 4-sulfation against malaria, analysis of the effects of lower ARSB and the associated increase in chondroitin 4-sulfation indicates increased susceptibility to malignant transformation and progression of malignancy, including in prostate, colon, mammary, and melanoma malignancies [7,12]. Analysis of ARSB immunohistochemical staining of human prostate cancer tissue microarrays with nearly 300 cases indicated that the decline in ARSB was associated with prostate cancers of a higher Gleason grade and with earlier recurrence [7]. Examination of human prostate tissues and cells showed that lower ARSB was associated with increased total sulfated glycosaminoglycans (GAGs) and chondroitin 4-sulfate (C4S) in malignant prostate tissues, malignant prostate cell lines, and prostate epithelial and stromal cells following ARSB knockdown by siRNA [7,12]. Declines in ARSB and CFTR have both been associated with increases in total sulfated glycosaminoglycans and chondroitin sulfate in human cells and tissues [6,7,12], consistent with an underlying biological mechanism linking defective CFTR and a decline in ARSB.

The data in this report provide additional evidence linking declines in CFTR and ARSB, focusing on the impact of decline in CFTR in normal human prostate epithelial cells on matrix metalloproteinases and other parameters of malignant progression. These data are presented in relation to findings in malignant human prostate tissues and cells and to the impact of ARSB knockdown and overexpression in prostate cells. The data which follow suggest that the occurrence of prostate cancer associated with defective CFTR may be related to a decline in ARSB and may be responsive to treatment by rhARSB.

## 2. Results

### 2.1. CFTR and ARSB in Normal and Malignant Human Prostate Tissue and Cells

**Figure 1:**
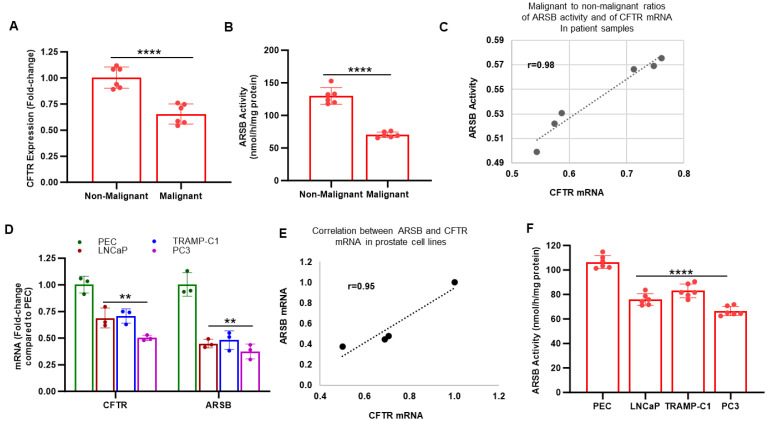
CFTR and ARSB in normal and malignant prostate tissue and cells. (**A**). CFTR expression is reduced in malignant human prostate tissue compared to associated non-malignant tissue (n = 6, *p* < 0.0001). (**B**). ARSB activity is reduced in the malignant prostate tissue (n = 6, *p* < 0.0001). (**C**). Correlation coefficient r equals 0.98 between the ARSB activity and CFTR expression in the prostate non-malignant and malignant tissues. (**D**). Both ARSB and CFTR mRNA expression are reduced in three malignant prostate cell lines (LNCaP, TRAMP-C1, and PC3), compared to the normal human prostate epithelial cells (PEC). All *p*-values are <0.01 (n = 6). (**E**). Correlation coefficient between ARSB activity and CFTR expression in the four cell lines in 1D is r equals 0.95. (**F**). ARSB activity is also reduced in the malignant cell lines, compared to the normal epithelial cells (n = 6, *p* < 0.00001). Two-tailed, unpaired *t*-tests, corrected for unequal variance, are used for calculation of *p*-values. ** represents *p* ≤ 0.01 and **** represents *p* ≤ 0.0001. [ARSB = arylsulfatase B, N-acetylgalactosamine-4-sulfatase; CFTR = cystic fibrosis transmembrane conductance regulator; PEC = prostate epithelial cells].

To detect if there was a relationship between CFTR and ARSB in human prostate tissue, tissue was obtained from surgeries for prostate cancer for measurements of CFTR and ARSB mRNA expression and ARSB activity. Normal and malignant tissues were isolated, and laser-capture microdissection was used to obtain distinct normal and malignant epithelial and stromal tissues [12]. CFTR expression was reduced in the malignant prostate tissue compared to the adjacent normal tissue (Figure 1A). ARSB mRNA and activity were reduced in the malignant prostate tissues (Figure 1B) [7,12]. The correlation coefficient between CFTR expression and ARSB activity is r = 0.98 (Figure 1C). Similar declines occurred for ARSB and CFTR mRNA expression in the malignant prostate cell lines LNCaP, TRAMPC1, and PC3, compared to expression in normal prostate epithelial cells (PEC) (Figure 1D). The correlation coefficient between CFTR mRNA and ARSB mRNA in these cell lines is r = 0.95 (Figure 1E). ARSB activity is also reduced in the malignant cell lines (*p* < 0.0001) (Figure 1F).

### 2.2. Effects of ARSB and CFTR Inhibition

**Figure 2:**
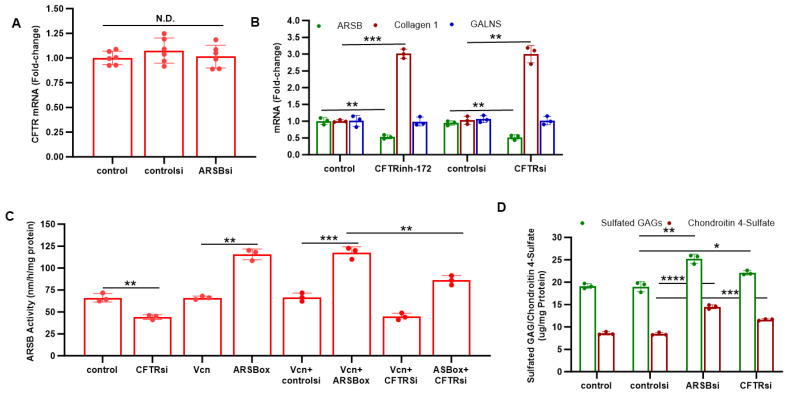
Effects of ARSB and CFTR inhibition. (**A**). Knockdown of ARSB by siRNA had no effect on CFTR mRNA expression in the normal PEC (n = 6). (**B**). Inhibition of CFTR by either the chemical inhibitor CFTRinh-172 or CFTR siRNA reduced ARSB expression (*p* < 0.01, n = 3), but had no impact on expression of GALNS (galactose 6-sulfate sulfatase) and significantly increased expression of Type 1 collagen (Collagen 1; COL1A2) (*p* = 0.008, *p* = 0.002). (**C**). When PC3 cells were treated with CFTR siRNA, ARSB activity declined (*p* = 0.006, n = 3). The effect of ARSB overexpression was inhibited by CFTR knockdown (*p* = 0.004, n = 3). (**D**). ARSB silencing increased total sulfated glycosaminoglycans (GAGs) and chondroitin 4-sulfate (C4S) (*p* = 0.002, 0.0001, n = 3) in the normal prostate epithelial cells. Similarly, following CFTR silencing, total sulfated (GAG) and C4S increased (*p* = 0.03, *p* = 0.0004, n = 3), consistent with inhibition of ARSB when CFTR is silenced. * represents *p* ≤ 0.05; ** is for *p* ≤ 0.01, *** for *p* ≤ 0.001, and **** for *p* ≤ 0.0001. [ARSBox = arylsulfatase B overexpression; GAGs = glycosaminoglycans; N.D. = no difference; PEC = prostate epithelial cells; Vcn = vector control].

Experiments were performed to determine if CFTR inhibition reduced ARSB expression in the prostate cell lines. Although silencing ARSB by siRNA had no effect on CFTR expression in the normal PEC (Figure 2A), inhibition of CFTR by either the chemical inhibitor CFTRinh-172 or CFTR siRNA reduced ARSB expression (*p* < 0.01) (Figure 2B). This was not a generalized effect of CFTR inhibition, since mRNA expression of collagen 1 (COL1A2) increased and expression of GALNS (galactose 6-sulfate sulfatase) did not change. When PC3 cells were treated with CFTR siRNA, ARSB activity declined (*p* < 0.01) (Figure 2C). The increase in ARSB activity induced by ARSB overexpression was reduced when CFTR was silenced (Figure 2C).

The impact of knockdown of ARSB and CFTR on total sulfated GAGs and on C4S was measured in the normal PEC. Both ARSB siRNA and CFTR siRNA significantly increased the total sulfated GAGs and C4S in the PEC, consistent with the impact of CFTR inhibition on ARSB activity (Figure 2D). ARSB silencing led to greater increases in sulfated GAGs and C4S than CFTR knockdown.

### 2.3. Inhibition of CFTR Replicates Effects of ARSB Knockdown on MMP2, MMP9 and Invasiveness

**Figure 3:**
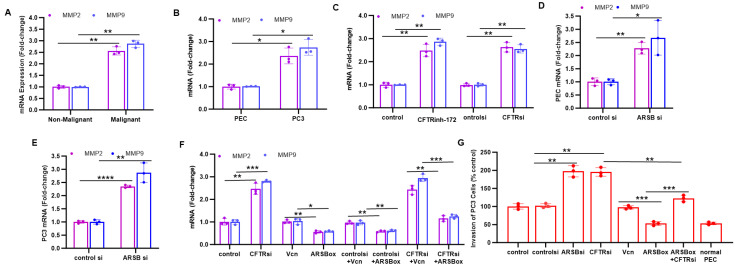
Inhibition of CFTR replicates effects of ARSB knockdown on MMP2, MMP9, and invasiveness. (**A**). Expression of MMP2 and MMP9 is significantly increased in malignant prostate tissue, compared to adjacent normal tissue (*p* = 0.002, *p* = 0.002; n = 3). (**B**). Expression of MMP2 and MMP9 is greater in the PC3 cells than in the normal PEC (*p* = 0.015, *p* = 0.013; n = 3). (**C**). Inhibition of CFTR by either CFTRinh-172 or siRNA in the PC3 cells increased the expression of MMP2 (*p* = 0.005, *p* = 0.003; n = 3) and MMP9 (*p* = 0.002, *p* = 0.002; n = 3). (**D**,**E**) ARSB silencing significantly increased MMP2 and MMP9 expression in the PEC cells (*p* = 0.002, *p* = 0.05; n = 3) and PC3 cells (*p* < 0.0001, *p* = 0.01; n = 3). (**F**). When ARSB was overexpressed in the PC3 cells, MMP2 and MMP9 expression declined (*p* = 0.0017, *p* = 0.026; n = 3). ARSB overexpression reduced the impact of CFTR silencing on MMP9 and MMP2 expression (*p* = 0.0016, *p* = 0.0005; n = 3). (**G**). Invasiveness of the PC3 cells increased when CFTR or ARSB was silenced (*p* = 0.0014, *p* = 0.004; n = 3). ARSB overexpression reduced the invasiveness to less than the baseline level in the PC3 cells (*p* = 0.0005; n = 3). The CFTR siRNA-induced increase in invasiveness was reduced when ARSB was overexpressed (*p* = 0.0014; n = 3). * represents *p* ≤ 0.05; ** is for *p* ≤ 0.01, *** for *p* ≤ 0.001, and **** for *p* < 0.0001. [ARSBox = ARSB overexpression; MMP = matrix metalloproteinase; PEC = prostate epithelial cells; si = siRNA; Vcn = vector control].

The impact of knockdown of CFTR and of ARSB on parameters of invasiveness was compared in the prostate tissue and cell lines. In malignant prostate tissue, expression of MMP2 and MMP9 was significantly greater than in the adjacent non-malignant tissue with higher ARSB expression (Figure 3A), indicating an inverse relationship between ARSB and MMP expression. Similarly, in the malignant PC3 cells, expression of MMP2 and MMP9 was significantly greater than in the normal PEC with higher ARSB (Figure 3B). When CFTR was inhibited by either CFTRinh-172 or by CFTR siRNA in the PC3 cells, expression of MMP2 and MMP9 increased (*p* < 0.01) (Figure 3C). ARSB siRNA increased expression of MMP2 and MMP9 in the PEC (Figure 3D) and PC3 (Figure 3E) to 2.3 and 2.7 times the control levels (Figure 3DE). When ARSB was overexpressed, MMP2 and MMP expression declined (Figure 3F). ARSB overexpression reduced the impact of CFTR silencing on MMP2 and MMP9 expression (Figure 3F). Consistent with the effects on MMP9 and MMP2 expression, invasiveness of the PC3 cells increased when CFTR or ARSB was silenced (Figure 3G). Inversely, ARSB overexpression reduced the invasiveness to less than the extent in the PC3 cells and to the level in the normal PEC and inhibited the CFTR siRNA-induced increase in invasiveness (Figure 3G).

### 2.4. Effects of ARSB and CFTR on c-Myc, EGFR, and BrdU Incorporation

**Figure 4:**
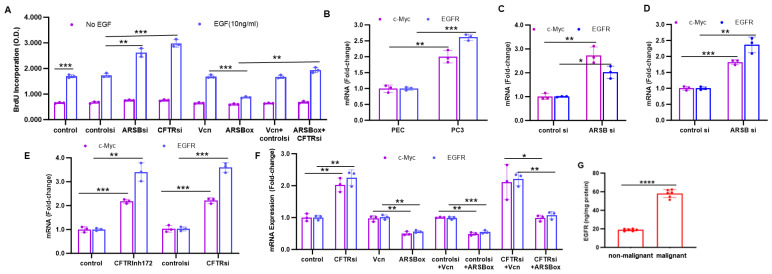
Effects of ARSB and CFTR on c-Myc and EGFR and BrdU incorporation. (**A**). EGF (10 ng/mL × 24 h) increased BrdU incorporation in the PC3 cells (*p* = 0.0004; *p* = 3). Both ARSB silencing and CFTR silencing further increased the response (*p* = 0.004, *p* = 0.0008; n = 3). ARSB overexpression reduced the BrdU incorporation (*p* = 0.0003; n = 3) and inhibited the effect of CFTR knockdown (*p* = 0.0009; n = 3). (**B**). In PC3 cells, mRNA expression of c-Myc and EGFR was greater than in normal PEC (*p* = 0.004, *p* = 0.00013; n = 3). (**C**). In the PEC, silencing ARSB increased mRNA expression of c-Myc (*p* = 0.004; n = 3) and EGFR (*p* = 0.00013; n = 3). (**D**). In PC3 cells, ARSB siRNA increased c-Myc (*p* = 0.008; n = 3) and EGFR (*p* = 0.02; n = 3). (**E**). In PC3 cells, CFTR inhibition by CFTRinh-172 or by siRNA significantly increased c-Myc (*p* = 0.0002, *p* = 0.0004; n = 3) and EGFR (*p* = 0.007, *p* = 0.0004; n = 3) expression. (**F**). In PC3 cells, CFTR siRNA significantly increased c-Myc (*p* = 0.005; n = 3) and EGFR (*p* = 0.0008; n = 3). ARSB overexpression reduced their expression (*p* = 0.0025, *p* = 0.0022; n = 3). In combination with CFTR silencing, ARSB overexpression reduced the CFTR knockdown-induced increases in c-Myc and EGFR (*p* = 0.066, *p* = 0.003; n = 3). (**G**). EGFR protein was significantly increased in the malignant compared to the adjacent non-malignant human prostate tissues (*p* < 0.0001; n = 6). * represents *p* ≤ 0.05; ** is for *p* ≤ 0.01, *** for *p* ≤ 0.001, and **** for *p* ≤ 0.0001. [ARSBox = ARSB overexpression; ARSBsi = ARSB siRNA; CFTRsi = CFTR siRNA; controlsi = control siRNA; EGF = epidermal growth factor; EGFR = epidermal growth factor receptor; PEC = prostate epithelial cells; Vcn = vector control].

Additional effects of ARSB and CFTR knockdown on other biologically significant parameters were detected and compared. The incorporation of 5-bromo-2′-deoxyuridine (BrdU) in response to EGF, CFTR silencing, ARSB silencing, and ARSB overexpression was measured in the PC3 cells (Figure 4A). EGF increased BrdU incorporation at 24 h in all groups, and both ARSB and CFTR silencing further increased the BrdU response (Figure 4A), reflecting enhanced proliferation. When ARSB was overexpressed, the BrdU incorporation in response to EGF declined (*p* < 0.001), and ARSB overexpression significantly reduced the effect of CFTR silencing on proliferation (*p* < 0.001).

In malignant PC3 cells, mRNA expression of c-Myc and EGFR was greater than in the normal PEC (Figure 4B), indicating increased expression in malignant cells. In the PEC (Figure 4C) and PC3 (Figure 4D) cells, ARSB silencing increased expression of c-Myc and EGFR. CFTR inhibition by CFTRinh-172 or by CFTR siRNA significantly increased c-Myc and EGFR expression in the PC3 cells (Figure 4E). In the PC3 cells, ARSB overexpression reduced the expression of c-Myc and EGFR (Figure 4F). When ARSB was overexpressed in combination with CFTR silencing, the CFTR knockdown-induced increases in c-Myc and EGFR were inhibited (Figure 4F). When total EGFR was measured in six malignant and associated non-malignant prostate tissues by ELISA, EGFR was 58.0 ± 4.2 ng/mg protein in the malignant tissue, compared to 18.9 ± 1.2 ng/mg protein in the non-malignant tissue (*p* < 0.0001), consistent with the measurements of ARSB and CFTR expression in the tissues (Figure 1A,B).

### 2.5. Impact of CFTR, Forskolin, and CREB on ARSB Expression

**Figure 5:**
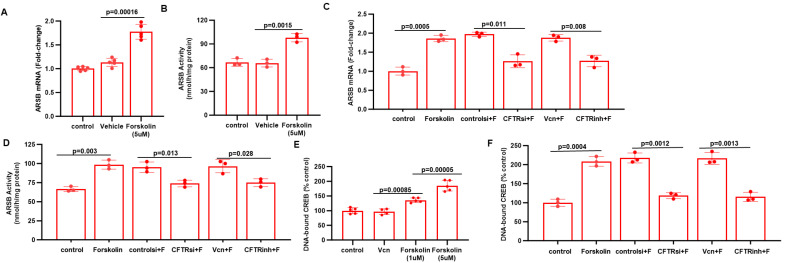
Impact of CFTR, forskolin, and CREB on ARSB expression. (**A**,**B**). Treatment of PC3 cells by forskolin (5 µM × 24 h) increased ARSB mRNA expression (*p* = 0.0002; n = 5) and activity (*p* = 0.0015; n = 3), consistent with a CFTR-mediated effect. (**C**,**D**). Inhibition of CFTR by siRNA or by CFTinh-172 blocked the forskolin-induced increases in ARSB expression (*p* = 0.011, *p* = 0.008; n = 3) and activity (*p* = 0.013, *p* = 0.028; n = 3). (**E**). Nuclear DNA-bound CREB (cAMP-responsive element binding protein) increased following forskolin (1 µM, *p* = 0.00085; 5 µM, *p* = 0.00005; compared to vehicle control; n = 5) and increased more with higher dose, indicating a dose-related increase. (**F**). The forskolin-induced increase in DNA-bound CREB was blocked by inhibition of CFTR by siRNA (*p* = 0.0012; n = 5) or CFTRinh-172 (*p* = 0.0013; n = 5). [CFTRinh = CFTRinh-172; controlsi = control siRNA; F = forskolin; Vcn = vehicle control].

To evaluate how CFTR might affect the expression of ARSB in the prostate cells, we tested the impact of forskolin, which activates adenyl cyclase leading to increased cAMP, PKA phosphorylation, and activation of CFTR. Experiments showed that treatment of PC3 cells by forskolin increased ARSB expression (*p* = 0.00016) (Figure 5A) and activity (*p* = 0.0015) (Figure 5B). The forskolin-induced increases in ARSB mRNA (Figure 5C) and activity (Figure 5D) declined when CFTR was inhibited by siRNA or by CFTRinh-172. DNA-bound CREB (cAMP-responsive element binding protein) increased following forskolin (Figure 5E). The increase in DNA-bound CREB declined when CFTR was inhibited (Figure 5F), suggesting that the forskolin-mediated increase in ARSB expression was due to CFTR.

## 3. Discussion

Prior publications demonstrated that a deficiency of CFTR was associated with a decline in ARSB expression and activity in human bronchial epithelial cells and in circulating neutrophils and mononuclear cells from patients with CF [6,7]. These findings provide a basis to account for the increase in chondroitin sulfates in bronchial secretions in patients with cystic fibrosis [6], since ARSB is required for the degradation of chondroitin 4-sulfate and dermatan sulfate. Since more sulfated chondroitin 4-sulfate (C4S) is associated with less adhesiveness of malarial-infected cells to placental and cerebral vasculature, a decline in ARSB has potentially served an evolutionary role in protection from malarial infection [9,10,11]. Defects in CFTR function might have provided a mechanism to reduce ARSB expression and activity and, thereby, increase chondroitin 4-sulfation and reduce malarial infectivity.

A specific mechanism for the decline in ARSB expression following a decline in CFTR may involve a decline in CREB when CFTR is defective, as suggested by the effects of forskolin and CFTR knockdown on ARSB expression and DNA-bound CREB in Figure 5. Moreover, a decline in ARSB activity might result from chloride-induced impairment of the formylglycine-modifying enzyme (FGE), which is required for the post-translational modification and activation of ARSB [8]. Furthermore, high chloride can reduce ARSB activity [7], suggesting that impaired chloride regulation by defective CFTR might directly inhibit ARSB in localized, cellular microenvironments.

Recent advances in treatment of CF by CFTR modifiers have markedly improved the quality of life and life expectancy of patients with CF. Since cancer incidence increases with age, the increased incidence of cancer in CF patients is becoming more apparent. Previous reports have shown predominantly increased colonic and pancreatic malignancies in CF, but increased prostate cancers have also been noted [1,2,3,4,5]. Reports have indicated that CFTR may act as a tumor suppressor in prostate cancers associated with bisphenol-A (BPA) exposure [13,14]. BPA is an endocrine disrupter and is frequently sulfated, suggesting interaction with overall pathways of cellular sulfate metabolism, which may be affected by both CFTR and ARSB [15,16,17]. Other intersections between the known effects of CFTR and of ARSB may help to elucidate complex biochemical pathways, environmental interactions, and cell signaling.

In the data presented in this short report, MMP2 and MMP9 expression and invasiveness of prostate cells were increased by either ARSB or CFTR inhibition. ARSB overexpression reduced the impact of CFTR inhibition on MMP2 and MMP9 expression, and on the invasiveness of PC-3 cells (Figure 3F,G). These findings are consistent with reports of increased MMP9 and MMP2 in CF patients compared to non-CF controls [18,19,20]. MMP9 was increased in plasma and in sputum of CF patients, and levels were inversely correlated with FEV_1_ [18,19]. Interestingly, lower serum MMP9 has been associated with responsiveness to treatment with CF modulators, suggesting that downregulation of MMP9 is a possible biomarker of treatment effectiveness [20]. Elevated MMPs may contribute to the airway remodeling which occurs in CF lungs. Other findings also demonstrate that a decline in CFTR by a specific inhibitor or by siRNA reduces ARSB and that ARSB overexpression can reduce the effect of CFTR inhibition on BrdU incorporation and on expression of EGFR and c-Myc, two proteins associated with malignancy. Overall, these data are consistent with an underlying mechanism whereby CFTR-induced decline in ARSB can contribute to malignancies associated with defective CFTR, and suggest that treatment with rhARSB may be beneficial in CF-associated malignancies.

## 4. Materials and Methods

### 4.1. Prostate Cell Lines and Tissue

Human prostate epithelial cells (PEC; ATCC^®^: CRL-2850^™^) were obtained and grown under the recommended conditions in Keratinocyte Serum Free Medium (K-SFM) with 0.05 mg/mL bovine pituitary extract (BPE) and 5 ng/mL epidermal growth factor (EGF). Cells were maintained at 37 °C in a humidified, 5% CO_2_ environment with replenishment of media every third day, as recommended. Confluent cells in T-25 flasks were harvested by EDTA-trypsin, and sub-cultured in multi-well tissue culture plates under similar conditions. Cells were grown to ~70–80% confluency, treated, and harvested by scraping or trypsinization [12]. Additional malignant prostate cell lines tested included: LNCaP (CRL-1740, ATCC, Manassas, VA, USA) from the lymph node of patient with metastatic prostate cancer; TRAMP-C1 (CRL-2730, ATCC) from the prostate of an adult male transgenic mouse with prostate adenocarcinoma; and PC-3 (CRL-1435, ATCC) from a bone metastasis of a patient with metastatic prostate cancer. These diverse cell lines were grown under the recommended conditions with media exchanges two to three times per week. Some cell preparations were treated with the CFTR inhibitor CFTR-I172 (#C2992, Sigma-Aldrich, St. Louis, MO, USA; 10 uM × 24 h), or forskolin (#S2449, Sigma-Aldrich; 1 uM or 5 uM × 24 h), or rhEGF (#236-EG, R&D, Minneapolis, MN, USA; 10 ng/mL × 24 h).

Fresh frozen tissues from nine radical prostatectomies performed for prostate cancer were obtained from the University of Illinois at Chicago (UIC) Biorepository under a protocol which was approved by the Institutional Review Board and the Cancer Center of UIC. Benign and associated malignant foci were isolated from frozen sections and identified in epithelium and stroma, dissected out, and frozen for subsequent analysis, as previously reported [12].

### 4.2. Laser Capture Microdissection

Normal and malignant prostate tissue samples from three patients were sectioned in a cryostat, and 7-micron-thick sections were placed on polyethylene naphthalate (PEN) membrane slides (Leica Microsystems, Buffalo Grove, IL, USA) [12]. Sections were cut by laser capture microdissection (LCMD) performed with Leica LMD 6000 laser dissection microscope with LMD software (version 6.5, Leica Microsystems). Sections were collected in LCMD-suitable collection vials (Thermo Fisher Scientific, Waltham, MA, USA) and promptly frozen at −80 °C.

### 4.3. ARSB and CFTR Silencing by siRNA

Specific siRNA for ARSB (EC 3.1.6.12) and control siRNA were procured (Qiagen, Germantown, MD, USA) and used, as previously described [7,12]. Specific siRNA for CFTR (#104323, Thermo Fisher Scientific, Waltham, MA, USA) was obtained and used for knockdown. Cells grew to 70% confluency in 12-well tissue culture clusters, and the medium of the growing cells was aspirated and replaced with 1.1 mL of fresh medium with serum. Then, 0.3 μL of 20 μM of siRNA (75 ng) was mixed with 100 μL of serum-free medium and 6 μL of HiPerfect Transfection Reagent (Qiagen). The mixture was incubated at room temperature for 10 min to allow the formation of transfection complexes and then added dropwise onto the cells. The plate was swirled gently, and treated cells were incubated at 37 °C in a humidified 5% CO_2_ environment. After 24 h, the medium was exchanged with fresh growth medium. The efficacy of the silencing procedure was determined by measurements of ARSB activity.

### 4.4. ARSB Overexpression

Overexpression of ARSB (NCBI NM_000046, transcript variant 1; TrueClone, OriGene, Rockville, MD, USA) was performed by transfection of specific untagged plasmids in pCMV6-XL4 vector using 2 μg of the plasmid and Lipofectamine^TM^ 2000 (Invitrogen, Carlsbad, CA, USA) [12]. Controls were cells transfected with vector only. Media were changed after 6 h, and cells were incubated for 48 h in a humidified, 37 °C, 5% CO_2_ environment, and then harvested.

### 4.5. ARSB Activity

ARSB activity in the control and treated prostate cells and tissue was determined as described [7]. Briefly, cells were harvested, and cell homogenates were prepared for measurement of ARSB activity. ARSB activity in the samples was determined using 4-methylumbelliferyl sulfate (MUS) as substrate in 0.05 M of acetate buffer, pH 5.6. ARSB activity was determined using a standard curve of known concentrations of methylumbelliferyl, and was expressed as nmol/mg protein/h.

### 4.6. Total Sulfated Glycosaminoglycan GAG and Chondroitin 4-Sulfate Assays

Total sulfated glycosaminoglycan (GAG) content in cell lysates was measured using the sulfated GAG assay (Blyscan^TM^, Biocolor Ltd., Newtownabbey, Northern Ireland), as previously described [7,12]. The sulfated polysaccharide component of the proteoglycans (PGs) and the protein-free sulfated GAG chains were detected, whereas degraded disaccharide fragments or hyaluronan was not. The reaction was performed in the presence of excess unbound dye (1, 9-dimethylmethylene blue). Triplicate 50 μL samples (containing < 5 μg of sulfated GAG and <250 mg cellular protein) were combined with 50 μL of deionized water in microcentrifuge tubes. Blyscan dye reagent (1.0 mL) was added, and tubes were mixed and shaken for 30 min. The cationic dye and GAG at acid pH produced an insoluble dye–GAG complex, forming a precipitate. The tubes were spun at 12,000 rpm for 10 min, and the unbound dye was removed. The Blyscan dissociation reagent (0.5 mL) was added, and the bound dye was mixed into solution. The mixture was centrifuged at 12,000 rpm for 5 min. Then, 200 μL of each sample were transferred to wells of a 96-well plate and absorbance was read at 656 nm, the absorbance maximum of 1,9-dimethylmethylene blue, in a microplate reader (FLUOStar, BMG, Cary, NC, USA). Readings were corrected by subtraction of the blank and compared to a standard curve prepared with known concentrations of sulfated GAGs. Concentration was expressed as micrograms/mg protein of cell or tissue lysate.

Chondroitin 4-sulfate monoclonal antibody (Clone LY111, AMS.A3143, Amsbio, Cambridge, MA, USA) is specific for the native chondroitin 4-sulfate, not the chondroitin stubs. Cell lysates were prepared from treated and control cells. Chondroitin 4-sulfate was immunoprecipitated from the cell lysates, as previously described [7,12]. The precipitate was eluted with dye-free elution buffer and subjected to sulfated GAG assay, as described above.

### 4.7. QRT-PCR for CFTR, EGFR, GATA-3, Collagen 1, ARSB, MMP2, MMP9, Cyclin-D1, c-Myc

Total RNA was prepared from malignant and normal prostate tissue and from control and treated prostate epithelial and stromal cells using RNeasy Mini Kit (Qiagen). QRT-PCR was performed as previously described [7,12] with specific primers. Each value was the mean of two determinations. Primers were:

ARSB (NM_000046) (forward): 5′-AGACTTTGGCAGGGGGTAAT-3′ and (reverse): 5′-CAGCCAGTCAGAGATGTGGA-3′;

CFTR (NM) (forward): 5′-GGAGAGCATACCAGCAGTGACT-3′ and (reverse): 5′-TTCCAAGGAGCCACAGCACAAC-3′;

EGFR: (NM_005228) (forward): 5′-AACACCCTGGTCTGGAAGTACG-3′ and (reverse): 5′-TCGTTGGACAGCCTTCAAGACC-3′;

GALNS (NM_000512) (forward): 5′-ACGGATTTGATGAGTGGTTTG-3′ and (reverse): 5′-GTAGAGGAAAAAGGGGTGGTG-3′;

GATA-3 (X55037): (forward): 5′-AGACCACCACAACCACACTCT-3′ and (reverse): 5′- GCCTTCCTTCTTCATAGTCAGG-3′;

c-Myc (NM_00246.7) (forward): 5′-GGAGGCTATTCTGCCCATTT-3′ and (reverse): 5′-AGGCTGCTGGTTTTCCACTAC-3′;

Cyclin D1 (CCND1; NM_053056.2) (forward): 5′-GATGGAGTTGTCGGTGTAGATG-3′ and (reverse): 5′-AACAGAAGTGCGAGGAGGAG-3′.

MMP2 (NM_004530) (forward): 5′-AGTGGATGATGCCTTTGCTC-3′ and (reverse): 5′-GAGTCCGTCCTTACCGTCAA-3′;

MMP9 (NM_004994.2) (forward): 5′-GTCTTCCCCTTCACTTTCCTG-3′ and (reverse): 5′-TCAGTGAAGCG GTACATAGGG-3′.

COL1A2 (NM_000089.3) (forward): 5′-AGCATCCATAGTGCATCCTTG-3′ and (reverse): 5′-TGGAGACTTCTACAGGGCTGA-3′;

Β-actin (NM_001101) (forward): 5′-GCCCCAAAAAGCAAAGATCA-3′ and (reverse): 5′-CCAGGAAGGAAGGCTGGAA-3′.

### 4.8. Total EGFR Assay

Total EGFR protein was measured in six non-malignant and associated malignant prostate tissues by ELISA (DYC1854, R&D, Biotechne, Minneapolis, MN, USA), following the recommended procedures. Data are expressed as ng/mg protein.

### 4.9. Nuclear Phospho-CREB Assay

Nuclear extracts were prepared from control and treated PC-3 or PEC cells using a commercial nuclear extract kit (#40410, Active Motif, Carlsbad, CA, USA). The nuclear cAMP response element-binding protein (CREB) was detected using an oligonucleotide-based transcription factor ELISA (#43096, Active Motif). Binding of nuclear CREB protein to the CREB oligonucleotide binding sequence (5′-TGACGTCA-3′) was detected using a specific CREB antibody. The extent of binding was detected using a secondary HRP-labeled antibody and measured at 450 nm in a microplate spectrophotometer (FLUOstar). Mutated CREB oligonucleotides were used as controls, and the extent of binding was expressed as % control.

### 4.10. BrdU Incorporation Assay

The BrdU incorporation assay was carried out using a commercial kit (ab126556, Abcam, Waltham, MA, USA), wherein 2 × 10^5^ cells/mL were plated in 100 μL/well in cell culture media. After 24 h, control silencing, CFTR siRNA, ARSB siRNA, and ARSB overexpression were carried out. At 48 h, 10 ng/mL of EGF was added to the experimental cells. Next, 20 μL of the diluted 1× BrdU was added to the wells, and plates were incubated for 24 h. Protocol was followed. The plate was read in a microplate spectrophotometer (FLUOstar) at 450 nm.

### 4.11. Invasion Assay

Cell invasiveness was detected using a fluorescent cell invasion assay (QCM^TM^ ECMatrix cell invasion assay, EMD 555, MilliporeSigma, Burlington, MA, USA). A 96-well cell culture plate and cell culture inserts with 8 μm pores in a polycarbonate membrane coated with a thin, dried layer of ECMatrix^TM^ to block non-invasive cell migration were used. The invasive cells migrated through the ECM layer and attached to the bottom of the polycarbonate membrane. The cells were dissociated from the membrane by incubation with Cell Detachment Buffer. Then, the invaded cells were lysed and detected by a green–fluorescent dye (CyQuant GR dye, Molecular Probes, Eugene, OR, USA) using a fluorescence plate reader (FLUOstar).

### 4.12. Statistics

Data presented are the mean ± SD of at least three independent experiments. Statistical significance was determined by unpaired *t*-test, two-tailed, corrected for unequal variance, using Microsoft Excel and Prism 10.1.2 software, unless stated otherwise. Specific data points are indicated, as well as mean value and standard deviation in the graphs. Correlation coefficient r is calculated using Microsoft Excel 365 software. *p* values ≤ 0.05 are considered statistically significant and are indicated by * for *p* ≤ 0.05, ** represents *p* ≤ 0.01, *** represents *p* ≤ 0.001, and **** represents *p* ≤ 0.0001.

## Data Availability

Data are available by communication with J.K.T.
